# STAT3 as a Candidate Shared Regulator of the CXCR4/CXCL12 and CXCR5/CXCL13 Homing Axes in Chronic Lymphocytic Leukemia

**DOI:** 10.3390/ijms27146099

**Published:** 2026-07-08

**Authors:** Aviwe Ntsethe

**Affiliations:** Department of Human Physiology, Nelson Mandela University, Gqeberha 6031, South Africa; aviwe.ntsethe@mandela.ac.za; Tel.: +27-41-504-4273

**Keywords:** chronic lymphocytic leukemia, CXCR4, CXCR5, CXCL12, CXCL13, STAT3, tumour microenvironment

## Abstract

Chronic lymphocytic leukemia (CLL) is characterised by the dependence of malignant cells on specialised tissue microenvironments within the bone marrow (BM) and secondary lymphoid organs (SLOs), which provide essential survival and proliferative signals. The CXCR4/CXCL12 and CXCR5/CXCL13 chemokine axes direct the trafficking of CLL cells into these anatomically distinct compartments, where stromal-derived survival signals protect them from both spontaneous and therapy-induced apoptosis. Although each chemokine axis has been extensively studied individually, no previous review has integrated both pathways into a unified mechanistic framework. This review proposes that the signal transducer and activator of transcription 3 (STAT3) function as a shared molecular hub that integrates niche-derived cytokine signals, including interleukin-6 (IL-6), IL-10, and IL-21, and may transcriptionally upregulate both CXCR4 and CXCR5, and reinforce tissue homing through a positive feedback loop. This review seeks to evaluate the expression, signalling, and clinical significance of each axis, their points of convergence and divergence and the therapeutic strategies that disrupt these parallel homing pathways. Complementing this framework, recent clinical evidence indicates that circulating CXCL13 serves as a robust prognostic biomarker in CLL, and that STAT3 inhibition may overcome bone marrow stromal-mediated cytoprotection. The CXCL12-CXCR4-STAT3-IL-10 immunosuppressive axis further drives T-cell exhaustion. Together, these pathways form an integrated oncogenic network that supports CLL cell survival, drives immune dysfunction, and promotes therapy resistance. Several important knowledge gaps remain. These include the lack of direct validation of the STAT3-CXCR5 transcriptional axis in primary CLL cells and uncertainty regarding whether CXCR4/CXCR5 dominance represents a stable transcriptional programme or a dynamic, microenvironment-driven process. Addressing these questions through single-cell transcriptomics, spatial transcriptomics, proteomics, and functional validation studies will be essential for developing rational combination therapies capable of simultaneously disrupting both homing axes.

## 1. Introduction

Chronic lymphocytic leukemia (CLL) is the most prevalent adult leukemia in Western countries, defined by the progressive accumulation of a mature, monoclonal CD5^+^CD19^+^CD23^+^ B-cell population in the peripheral blood, bone marrow (BM) and secondary lymphoid organs (SLO) [1,2]. While the vast majority of circulating CLL cells are arrested in the G0/G1 phase of the cell cycle and exhibit prolonged survival in vivo, these same cells undergo rapid spontaneous apoptosis within hours to days when placed in conventional cell culture [3,4]. This discordance between in vivo longevity and in vitro fragility reflects an obligate metabolic and signalling dependence on tissue-derived niches that cannot be replicated ex vivo.

Within the BM and SLOs, CLL cells receive multiple complementary survival signals. These include contact-dependent interactions with stromal cells through adhesion molecules such as very late antigen-4 (VLA-4), soluble mediators including B-cell activating factor (BAFF), a proliferation-inducing ligand (APRIL), IL-6, and additional cytokine-driven signalling pathways [5,6]. Collectively, these signals activate phosphatidylinositol 3′-kinase (PI3K)-AKT, nuclear factor (NF)-κB, and Janus Kinase (JAK)/signal transducer and activator of transcription 3 (STAT3) signalling, thereby promoting expression of anti-apoptotic B-cell lymphoma 2 (BCL-2) family proteins and resistance to therapy [7].

The tumour microenvironment (TME) in CLL encompasses bone marrow mesenchymal stromal cells (BMSCs), nurse-like cells (NLCs), follicular dendritic cells (FDCs), and a variety of soluble mediators including cytokines, chemokines, and immune checkpoint ligands [3,4,8,9]. Together these create a permissive niche that protects CLL cells from apoptosis, drives proliferation within pseudofollicular proliferation centres, and actively suppresses host anti-tumour immunity [10].

Chemokine receptors are seven-transmembrane G protein-coupled receptors (GPCRs) whose principal physiological role in the B-cell lineage is the co-ordination of spatiotemporal positioning during development and immune activation. CXCR4 and its non-redundant ligand CXCL12, also known as stromal cell-derived factor-1 (SDF-1), are essential for normal B lymphopoiesis. The genetic deletion of either Cxcr4 or Cxcl12 in mice results in a severe reduction in BM B-cell precursors [11,12]. In mature B cells, CXCR4 mediates recirculation through the BM sinusoids and participates in plasmablast retention at plasma cell survival niches [13].

CXCR5, which binds the homeostatic chemokine CXCL13, plays a complementary and anatomically distinct role. In secondary lymphoid organs, FDCs and stromal cells constitutively secrete CXCL13 to form a chemokine gradient that recruits naive B cells from the T-cell zone into follicles and thereafter positions antigen-activated B cells within the light zone of the germinal centre (GC) for affinity maturation. CXCR5 is additionally expressed on follicular helper T (Tfh) cells, facilitating their co-localisation with GC B cells [14,15]. The reciprocal downregulation of CXCR5 and upregulation of CXCR4 during the transition from naive B cell to plasma cell illustrates how these two receptors operate as opposing positional signals [16].

In CLL, the normal developmental regulation of CXCR4 and CXCR5 is co-opted by the malignant clone, which exploits both receptors simultaneously to access parallel survival niches. The CXCR4/CXCL12 axis directs CLL cells to BM stromal microenvironments enriched with pro-survival factors and hypoxic conditions that favour quiescence and cell adhesion-mediated drug resistance (CAM-DR) [17,18,19]. The CXCR5/CXCL13 axis, by contrast, mediates homing to lymph node (LN) follicles and the formation of pseudofollicles, the specialised proliferative compartments in which T-cell help and B-cell receptor (BCR) co-stimulation drive clonal expansion [20]. These two niches fulfil distinct but complementary functions; the BM niche primarily protects cells from apoptosis and drug exposure, while the LN niche provides the proliferative stimulus that sustains tumour mass over time.

Despite this functional complementarity, the two axes have rarely been studied in an integrated framework. Most published work has examined CXCR4 or CXCR5 in isolation, addressing expression, signalling, or clinical correlates of one axis without reference to the other. This has led to an unanswered question: To what extent do CLL cells dynamically redistribute between BM and LN compartments according to the relative availability of CXCL12 and CXCL13, and what molecular mechanisms establish this receptor hierarchy? The present review aims to address this question within a unified biological and clinical framework.

STAT3 is a latent cytoplasmic transcription factor activated by a broad array of extracellular stimuli through canonical JAK-dependent and non-canonical Src/ABL-dependent pathways [21]. In CLL, STAT3 occupies a pivotal position at the intersection of multiple microenvironmental inputs. Tyrosine phosphorylation at Tyr705 (pY705) that is required for STAT3 dimerisation, nuclear translocation, and full transcriptional activity is predominantly induced within tissue niches by cytokines such as IL-6, IL-10, and IL-21, as well as by BCR engagement acting through JAK2 [22,23,24].

Severin et al. [25] provided detailed mechanistic characterisation of JAK2/STAT3 signalling in CLL, demonstrating significantly higher protein expression of both STAT3 and JAK2 in CLL cells compared to normal B lymphocytes across a cohort of 66 treatment-naive patients. Constitutive Tyr705-STAT3 phosphorylation was demonstrated in patients with CLL, and Tyr705-STAT3 levels correlated with clinical aggressiveness, cytopenias, and advanced Rai stage [25].

The transcriptional targets of activated STAT3 in CLL include the anti-apoptotic protein BCL-2, the immunosuppressive cytokine IL-10, and the chemokine receptors CXCR4 and CXCR5 themselves [26]. If STAT3 directly upregulates both migratory receptors, cytokine-driven STAT3 activation within niches would not only enhance survival but would also deepen tissue homing, a positive feedback that makes STAT3 a uniquely attractive therapeutic target.

This review systematically examines the CXCR4/CXCL12 and CXCR5/CXCL13 axes in CLL, with particular focus on their downstream signalling, clinical correlates, transcriptional regulation by STAT3, and therapeutic relevance. A literature search was conducted in PubMed (MEDLINE), EBSCOhost (Academic Search Complete database) using the terms “CLL”, “chronic lymphocytic leukemia”, “CXCR4”, “CXCR5”, “CXCL12”, “CXCL13”, “STAT3”, “JAK2” and relevant combinations thereof from inception to 15 April 2026. Study selection primarily focused on studies published during the last 5 years to capture the most recent developments; however, older foundational studies were also included where they provided essential mechanistic or contextual insight. Additional searches were performed using Google Scholar to identify supplementary articles.

## 2. Literature Review

### 2.1. The CXCR4/CXCL12 Axis: Bone Marrow Homing

#### 2.1.1. Expression of CXCR4 on Malignant Cells Versus Normal B Cells

Surface CXCR4 is constitutively overexpressed on malignant B cells compared with normal peripheral blood B lymphocytes. Quantitative flow cytometric analyses report a 5- to 22-fold increase in mean fluorescence intensity (MFI), depending on the antibody clone and gating strategy employed [27]. This overexpression is independent of classical adverse prognostic markers including ZAP-70 positivity and unmutated immunoglobulin heavy-chain variable region gene (IGHV) status, suggesting that elevated CXCR4 is a fundamental characteristic of the CLL phenotype rather than a correlate of genomic risk [27].

Importantly, surface CXCR4 is dynamically regulated. CLL cells isolated from bone marrow biopsies express lower surface receptor levels than paired circulating counterparts, consistent with ligand-induced internalisation within the CXCL12-rich marrow environment [28,29]. The CXCR4 gene encodes two splice variants, an isoform A (CXCR4a), which predominates in lymphoid cells, and isoform B (CXCR4b), which has been more extensively studied in HIV co-receptor biology [30].

#### 2.1.2. CXCL12 Signalling in the Bone Marrow Niche

Within the bone marrow, CXCL12 is constitutively produced by a specialised hierarchy of stromal cell populations. CXCL12-abundant reticular (CAR) cells, a distinct subset of CXCL12^high^CXCR4^high^ reticular cells located adjacent to the endosteum and vascular sinusoids, represent the principal source of CXCL12 within the marrow [31]. Bone marrow mesenchymal stromal cells (BMSCs) and mesenchymal stem cells (MSCs) also secrete physiologically significant amounts of CXCL12. Alternative splicing of the CXCL12 gene generates at least six isoforms, of which CXCL12α and CXCL12β are the predominant forms expressed within the bone marrow [32].

CXCL12 exerts its biological effects through two receptors, CXCR4 (CD184) and atypical chemokine receptor 3 (ACKR3; also known as CXCR7). CXCR4 is a classical G protein-coupled receptor that activates Gαi proteins following ligand binding, resulting in inhibition of adenylyl cyclase and activation of multiple downstream signalling pathways, including PI3K/AKT, MEK/ERK1/2, and JAK2/STAT3 [33,34,35]. In contrast, ACKR3 functions primarily as a β-arrestin-biased receptor and preferentially signals through AKT and ERK pathways, thereby indirectly enhancing STAT3 transcriptional activity [36].

Structural studies have demonstrated that the N-terminal region of the third intracellular loop (ICL3) of CXCR4 is essential for JAK2 activation. Activated JAK2 subsequently phosphorylates Tyr157 within the second intracellular loop (ICL2), thereby initiating downstream STAT3 signalling [37].

CXCL12 production within the bone marrow microenvironment is further enhanced under hypoxic conditions through hypoxia-inducible factor-1α (HIF-1α)-mediated transcriptional activation of the CXCL12 promoter [38,39]. This hypoxia-driven increase in CXCL12 establishes a self-reinforcing retention mechanism. Cells recruited into hypoxic niches encounter progressively higher CXCL12 concentrations, promoting sustained CXCR4-mediated retention while simultaneously increasing CXCR4 expression through HIF-1α-dependent transcriptional regulation.

#### 2.1.3. Downstream Signalling of CXCR4 in CLL

CXCR4 is a pertussis toxin-sensitive Gαi-coupled G protein-coupled receptor (GPCR). Binding of CXCL12 induces dissociation of the Gαi and Gβγ subunits, resulting in inhibition of adenylyl cyclase and activation of several downstream signalling pathways [33,34,35]. Among these, the PI3K/AKT pathway represents one of the principal mediators of CXCR4 signalling in CLL. Activation of PI3Kγ and PI3Kδ by Gβγ subunits generates phosphatidylinositol (3,4,5)-trisphosphate (PIP3), which subsequently activates AKT. Activated AKT phosphorylates and inactivates the pro-apoptotic protein BCL-2-associated death promoter (BAD) while simultaneously inhibiting FOXO transcription factors, thereby suppressing pro-apoptotic gene expression and promoting CLL cell survival [35].

Beyond PI3K/AKT signalling, CXCL12-mediated activation of CXCR4 stimulates the JAK2/STAT3 pathway. Engagement of CXCR4 induces phosphorylation of STAT3 at Ser727, which is sufficient to promote transcription of IL-10 and other immunosuppressive genes, even in the absence of Tyr705 phosphorylation [22]. CXCR4-mediated signalling also activates AKT and c-Jun N-terminal kinase (JNK), leading to increased IL-6 secretion. IL-6 subsequently reinforces STAT3 activation through the JAK/STAT3/Snail signalling axis, thereby establishing a positive feedback loop that sustains tumour-promoting signalling. Conversely, activation of the CXCL12/CXCR4 axis induces suppressor of cytokine signalling 3 (SOCS3), which binds CXCR4 and negatively regulates both Gi-mediated and JAK/STAT signalling, providing an important feedback mechanism that limits excessive pathway activation [25]. CXCR4 signalling is further amplified through cross-talk with BCR signalling. Bruton’s tyrosine kinase (BTK), a critical component of the BCR pathway, enhances CXCR4-mediated migration and tissue homing, thereby integrating chemokine receptor signalling with antigen receptor activation.

An important but often overlooked aspect of CXCR4 signalling is its marked heterogeneity within the malignant clone. Rather than representing a homogeneous population, circulating CLL cells comprise functionally distinct subpopulations that reflect their recent trafficking history. Calissano et al. [40] identified two intraclonal populations characterised by reciprocal expression of CXCR4 and CD5: a proliferative CXCR4^dim^CD5^bright^ fraction and a quiescent CXCR4^bright^CD5^dim^ fraction.

The CXCR4^dim^CD5^bright^ population is enriched for recently divided lymph node emigrants and exhibits a transcriptional programme characterised by increased proliferation, enhanced survival signalling, and elevated expression of anti-apoptotic genes. This population also expresses high levels of CCR7, consistent with recent exposure to tissue-derived chemokine gradients and ligand-induced CXCR4 internalisation [40,41]. In contrast, the CXCR4^bright^CD5^dim^ fraction represents older, quiescent cells that are primed either to re-enter supportive tissue niches or to undergo apoptosis.

Bartholdy et al. [42] further demonstrated that these subpopulations possess distinct DNA methylation landscapes reflecting their proliferative histories. The proliferative CXCR4^dim^ fraction exhibited enrichment of epigenetic signatures associated with disease progression. Collectively, these findings suggest that CXCR4 surface expression should not be regarded solely as a marker of chemotactic responsiveness but rather as a dynamic indicator of niche exposure, signalling activity, epigenetic programming, and clonal fitness.

The biological significance of the proliferative fraction is reinforced by its preferential responsiveness to IL-23. Cardillo et al. [43] demonstrated that IL-12Rβ1 expression is significantly higher in proliferating CLL cells than in resting cells and that stimulation preferentially induces formation of the IL-23 receptor complex rather than the IL-12 receptor complex. Because IL-23 activates JAK2/TYK2-dependent STAT3 signalling, preferential IL-23 responsiveness may further amplify STAT3 activation within the recently divided cell population, thereby reinforcing tissue-derived survival signals.

#### 2.1.4. CXCR4-STAT3-IL-10: CLL-Mediated Immunosuppression

A defining feature of CLL is the establishment of an immunosuppressive microenvironment that promotes tumour persistence while impairing anti-tumour immunity. A critical component of this process is the CXCL12-CXCR4-STAT3-IL-10 signalling axis, which directly links tissue homing to immune evasion.

Shaim et al. [26] demonstrated that stimulation of primary CLL cells with CXCL12 significantly increased phosphorylation of STAT3 at Ser727. This effect was abolished by both CXCR4 blockade and pharmacological inhibition of STAT3 using cucurbitacin, confirming that STAT3 activation occurs downstream of CXCR4 signalling. Consistent with these findings, CXCL12 stimulation markedly increased IL-10 production by CLL cells, whereas inhibition of CXCR4 or STAT3, or STAT3 knockdown using short hairpin RNA (shRNA), significantly reduced IL-10 secretion.

The functional consequences of this signalling pathway were demonstrated using co-culture experiments. CXCL12 markedly enhanced the capacity of CLL cells to suppress CD3^+^ T-cell effector functions, including production of tumour necrosis factor-α (TNF-α), interferon-γ (IFN-γ), interleukin-2 (IL-2), and CD107a-mediated degranulation. Importantly, this immunosuppressive effect was completely reversed by either CXCR4 blockade or IL-10 neutralisation, demonstrating that IL-10 is the principal downstream mediator of CXCL12-induced T-cell suppression [26].

Collectively, these findings support a two-step immunosuppressive model. First, CXCL12 activates CXCR4 on CLL cells, inducing STAT3 Ser727 phosphorylation and IL-10 secretion. Subsequently, IL-10 acts on neighbouring T cells through the IL-10 receptor, triggering Tyr705 phosphorylation of STAT3 and ultimately suppressing T-cell effector function. This signalling cascade establishes a mechanistic link between chemokine-directed tissue homing and immune escape, thereby contributing to disease progression and therapeutic resistance.

#### 2.1.5. CXCR4 and Pseudoemperipolesis

Pseudoemperipolesis (PEP), defined as the spontaneous migration of CLL cells beneath or into the cytoplasm of bone marrow stromal cells, represents one of the hallmark interactions between CLL cells and the bone marrow microenvironment [19]. This process is critically dependent on coordinated activation of the CXCR4/CXCL12 axis and adhesion molecules, particularly VLA-4, which interacts with vascular cell adhesion molecule-1 (VCAM-1) expressed on stromal cells [44,45]. CXCR4-mediated signalling promotes pseudopod formation through activation of the PI3K pathway, whereas VLA-4 mediates firm adhesion to stromal cells. Together, these mechanisms facilitate migration beneath the stromal layer, where CLL cells receive potent anti-apoptotic signals that protect them from chemotherapeutic agents, including fludarabine and chlorambucil.

Pharmacological inhibition of CXCR4 effectively disrupts pseudoemperipolesis both in vitro and in vivo, resulting in mobilisation of CLL cells from the protective bone marrow niche into the peripheral circulation [17,27,46]. These findings demonstrate that CXCR4 is not only a chemotactic receptor but also a critical mediator of physical interactions between CLL cells and the stromal microenvironment.

#### 2.1.6. CXCR4 Expression as a Clinical Biomarker

Surface CXCR4 expression increases progressively with disease stage. Patients with Rai stage IV disease consistently exhibit significantly higher receptor expression than those with early-stage disease [47]. Furthermore, CXCR4 expression correlates positively with absolute lymphocyte count and the extent of bone marrow infiltration [45,48], supporting its role as a marker of disease burden. CXCR4 has been proposed as an independent predictor of shorter progression-free survival (PFS) in multivariate analysis, though this requires prospective validation [49]. The relationship between CXCR4 expression and other prognostic markers such as zeta-chain-associated protein kinase 70 (ZAP-70), CD38, IGHV mutation status is nuanced, with some studies reporting positive correlations and others finding no significant association after adjusting for clinical stage [26,27,47,49,50].

The most comprehensive prospective evaluation of CXCR4 as a prognostic biomarker was performed by Xue et al. [49], who quantified CXCR4 surface expression at diagnosis in patients with untreated CLL. Patients with high CXCR4 expression experienced significantly shorter PFS during a median follow-up of 27 months. Importantly, multivariable Cox regression demonstrated that CXCR4 overexpression remained an independent predictor of disease progression after adjustment for established prognostic variables, including lymphocyte count, platelet count, haemoglobin concentration, lactate dehydrogenase, CD38 expression, and Rai stage. These findings suggest that CXCR4 provides prognostic information that complements conventional clinical and laboratory markers.

Collectively, the available evidence supports CXCR4 overexpression as a biologically meaningful marker of disease aggressiveness [49,51,52]. However, inconsistencies between studies, including differences in patient populations, sample sources, follow-up duration, and flow cytometric methodologies, currently limit its routine clinical implementation [49,51,53]. Standardised multicentre prospective studies incorporating comprehensive molecular profiling will be required before CXCR4 can be incorporated into risk stratification algorithms or clinical decision-making. Given accumulating evidence implicating STAT3 in CXCR4 transcription, future biomarker studies should evaluate CXCR4 expression together with STAT3 activation status. Such integrated analyses may improve prognostic accuracy while simultaneously identifying patients most likely to benefit from therapies targeting the STAT3-CXCR4 signalling axis.

#### 2.1.7. Transcriptional and Post-Translational Regulation of CXCR4

**Figure 1:**
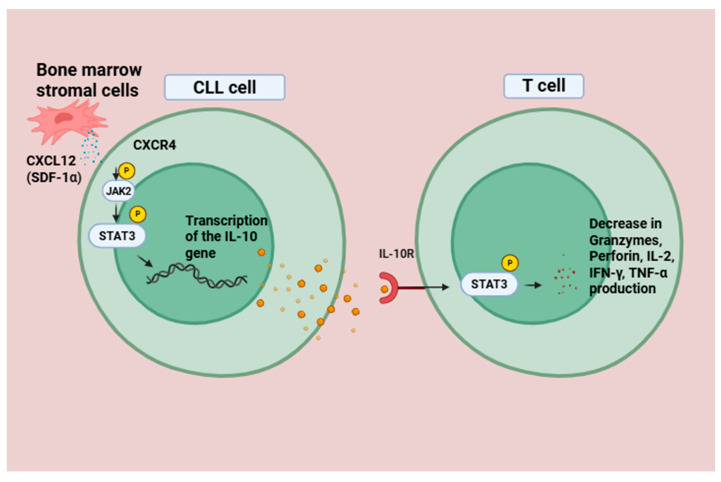
**CXCL12–CXCR4–STAT3–IL-10 signalling axis mediating immune suppression in chronic lymphocytic leukemia**. Bone marrow stromal cells secrete CXCL12 (SDF-1α), which binds to CXCR4 on CLL cells and activates downstream signalling pathways, including JAK2 and STAT3. CXCR4 activation promotes phosphorylation of STAT3 (primarily pS727-STAT3), leading to nuclear translocation of STAT3 and transcription of the IL-10 gene. Secreted IL-10 then acts on T cells through the IL-10 receptor (IL-10R), inducing phosphorylation of STAT3 at Y705 and suppressing T-cell effector functions, including TNF-α, IFN-γ, IL-2 production, and cytolytic degranulation (CD107a) [26]. Created in BioRender, https://BioRender.com (accessed on 23 May 2026).

Regulation of CXCR4 expression occurs at both transcriptional and post-translational levels, allowing CLL cells to dynamically adapt receptor abundance in response to microenvironmental signals [39]. Under hypoxic conditions, HIF-1α drives robust transcriptional upregulation of CXCR4, synergising with concomitant HIF-1α-dependent induction of CXCL12 in stromal cells [39]. STAT3 inhibition reduces this hypoxia-driven response, implicating a HIF-1α-STAT3 cooperative axis at the CXCR4 promoter [39]. STAT3 can also bind the CXCL12 promoter directly to enhance ligand expression in stromal cells, suggesting that STAT3 may reinforce the CXCR4/CXCL12 axis by augmenting ligand availability within the niche itself (Figure 1) [26].

Collectively, these findings position STAT3 as a central regulatory node linking hypoxia, inflammatory cytokine signalling, and chemokine receptor expression. Rather than acting solely downstream of CXCR4 activation, STAT3 appears capable of reinforcing the entire CXCR4/CXCL12 signalling axis by simultaneously promoting receptor expression, increasing ligand availability, and sustaining survival signalling within the bone marrow microenvironment. This positive feedback mechanism provides the conceptual foundation for the central hypothesis proposed in this review.

### 2.2. The CXCR5/CXCL13 Axis: Lymph Node Colonisation and Pseudofollicle Formation

#### 2.2.1. CXCR5 Expression on CLL Cells

CXCR5 is highly expressed on CLL cells and plays a central role in directing their migration into lymphoid tissues. Quantitative flow cytometric analyses have demonstrated markedly higher CXCR5 surface expression on CLL cells than on normal B lymphocytes and Tfh cells [54]. These findings indicate that CXCR5 overexpression is a characteristic feature of the CLL phenotype.

In contrast to CXCR4, whose expression increases with disease progression, CXCR5 expression remains relatively stable across Rai and Binet clinical stages and shows little association with IGHV mutation status or CD38 expression [54,55]. This stability suggests that CXCR5 dysregulation occurs early during leukemogenesis and is subsequently maintained throughout disease progression.

Exposure of CLL cells to exogenous CXCL13 or prolonged co-culture with NLCs results in marked receptor internalisation and reduced surface expression, consistent with ligand-dependent receptor recycling within the lymph node microenvironment [54]. These observations indicate that CXCR5, like CXCR4, undergoes dynamic regulation in response to its ligand despite its relatively stable baseline expression.

#### 2.2.2. CXCL13 in the CLL Lymph Node Microenvironment

Within lymph nodes infiltrated by CLL cells, CXCL13 is produced predominantly by NLCs, FDCs, and Tfh cells [54,56]. Together, these cellular populations establish a specialised microenvironment that promotes tumour cell recruitment, survival, and proliferation.

NLCs play a particularly important role by simultaneously producing CXCL13, BAFF, APRIL, and additional co-stimulatory mediators that collectively support CLL cell survival [56]. In parallel, interactions between CLL cells and stromal cells through lymphotoxin-β receptor (LTβR) signalling stimulate further CXCL13 production, thereby reinforcing lymph node retention and establishing a self-sustaining positive feedback loop [57,58].

Heinig et al. [58] demonstrated that CLL cells actively remodel their own microenvironment. Through expression of lymphotoxin-αβ, malignant cells activate LTβR signalling on stromal cells and follicular dendritic cells, inducing CXCL13 secretion that subsequently promotes additional tumour cell recruitment and retention. Consequently, CXCR5 signalling not only directs lymph node homing but also contributes to the construction and maintenance of the supportive tumour niche.

Beyond regulating migration, CXCL13 activates ERK1/2 and AKT/GSK3 signalling pathways, suppresses FOXO3a-mediated apoptosis, and promotes long-term survival of CLL cells [20]. The reciprocal nature of this CXCL13 gradient has been clarified mechanistically by Heinig et al. [58] who demonstrated that CLL B cells activate LTβR through lymphotoxin-αβ production, inducing the very CXCL13 gradient that sustains their own pseudofollicular retention. This autocrine-paracrine loop means CXCR5 activity simultaneously directs tumour cell homing, constructs the survival niche, and enables FDC-derived proliferation stimuli. Bürkle et al. [54] further demonstrated that CXCL13 stimulation induces actin polymerisation, receptor internalisation, and MAPK activation, thereby enhancing both migratory capacity and resistance to apoptosis. Importantly, sustained ERK activation provides a plausible mechanistic link to STAT3 through phosphorylation of STAT3 at Ser727. Although this signalling pathway has not yet been fully characterised in primary CLL cells, it suggests that CXCR5 may converge upon STAT3 independently of the classical CXCR4–JAK2 signalling pathway.

#### 2.2.3. CXCL13 as a Prognostic Biomarker in CLL

Growing evidence supports circulating CXCL13 as a clinically relevant biomarker of disease activity in CLL. Multiple studies have demonstrated that elevated plasma or serum CXCL13 concentrations are associated with advanced clinical stage, increased lymph node tumour burden, enhanced proliferative activity of CLL cells, and inferior clinical outcomes [20,59].

Sivina et al. [20] first demonstrated that plasma CXCL13 concentrations correlate with lymphadenopathy and decline substantially during successful ibrutinib therapy before increasing again in patients who develop treatment resistance. These observations suggest that CXCL13 reflects dynamic changes within the lymph node microenvironment and may therefore serve as a pharmacodynamic biomarker of tissue disease.

These findings were subsequently strengthened by Ahmed et al. [59], who prospectively followed 91 treatment-naïve patients with CLL for up to 54 months. Patients with elevated plasma CXCL13 concentrations experienced significantly shorter PFS and time to first treatment (TTFT). Furthermore, increased CXCL13 levels were associated with adverse prognostic characteristics, including del(17p), elevated β2-microglobulin concentrations, CD38 positivity, and advanced Rai stage.

Collectively, these studies indicate that CXCL13 reflects both biological activity within the lymph node microenvironment and overall disease aggressiveness. Nevertheless, current evidence remains limited to relatively small patient cohorts, and larger prospective multicentre studies are required before CXCL13 can be incorporated into routine clinical risk stratification.

#### 2.2.4. Combined CXCL13 and Galectin-9 as Collaborative Biomarkers

Although CXCL13 primarily reflects lymph node homing and microenvironmental activity, combining CXCL13 with biomarkers of immune dysfunction may improve disease stratification. One promising candidate is galectin-9 (Gal-9), an immunoregulatory lectin that contributes to T-cell exhaustion through interaction with T-cell immunoglobulin and mucin-domain containing-3 (Tim-3).

Ahmed et al. [59] demonstrated that concurrent measurement of CXCL13 and galectin-9 improved discrimination between patients with CLL and healthy controls and enhanced identification of patients with high-risk disease. These complementary biomarkers capture distinct aspects of CLL biology: CXCL13 reflects tumour–microenvironment interactions, whereas galectin-9 reflects immune suppression.

Although galectin-9 is not directly involved in chemokine-mediated homing, its association with CXCL13 highlights the close relationship between tissue localisation and immune dysfunction within the CLL microenvironment.

#### 2.2.5. Downstream Signalling of CXCR5 in CLL

Following engagement by CXCL13, CXCR5 activates several intracellular signalling pathways that promote migration, survival, and proliferation of CLL cells. Among these, PI3Kδ is the dominant downstream signalling mediator. Pharmacological inhibition of PI3Kδ with idelalisib effectively suppresses CXCL13-induced chemotaxis, demonstrating the importance of this pathway in lymph node homing [60].

In contrast to many chemokine receptors that induce transient extracellular signal-regulated kinase (ERK) activation, CXCR5 stimulation produces sustained ERK1/2 phosphorylation in CLL cells [61]. Persistent ERK activation is likely to support prolonged transcriptional programmes associated with lymph node retention, cellular survival, and resistance to apoptosis.

CXCR5-mediated localisation within pseudofollicles also facilitates direct interactions between CLL cells and activated CD4^+^ T cells. These interactions provide CD40 ligand (CD40L) and IL-4, activating NF-κB and STAT6 signalling pathways that further promote tumour cell survival [23,62,63].

Beyond effects on malignant B cells, CXCR5 signalling contributes to remodelling of the immune microenvironment. Dreisinger et al. [64] demonstrated that CXCR5^+^ T follicular regulatory (Tfr-like) cells are increased in patients with Binet stage B CLL and correlate with tumour burden and lymphadenopathy. These observations suggest that CXCR5 signalling contributes not only to tumour cell localisation but also to the establishment of an immunosuppressive lymph node microenvironment.

Mechanistically, Foster et al. [65] demonstrated that cooperative STAT3 and SMAD signalling promotes BCL6 induction during Tfh cell differentiation, resulting in acquisition of CXCR5, PD-1, and IL-21 expression. Although these findings were generated outside the CLL setting, they provide indirect mechanistic support for a potential interaction between STAT3 signalling and the CXCR5 homing programme.

Collectively, the available evidence indicates that CXCR5 signalling integrates chemotaxis, survival, and immune regulation within the lymph node microenvironment. However, whether STAT3 directly regulates CXCR5 expression or primarily influences downstream biological responses remains unresolved in primary CLL cells.

#### 2.2.6. Transcriptional Regulation of CXCR5 in CLL

**Figure 2:**
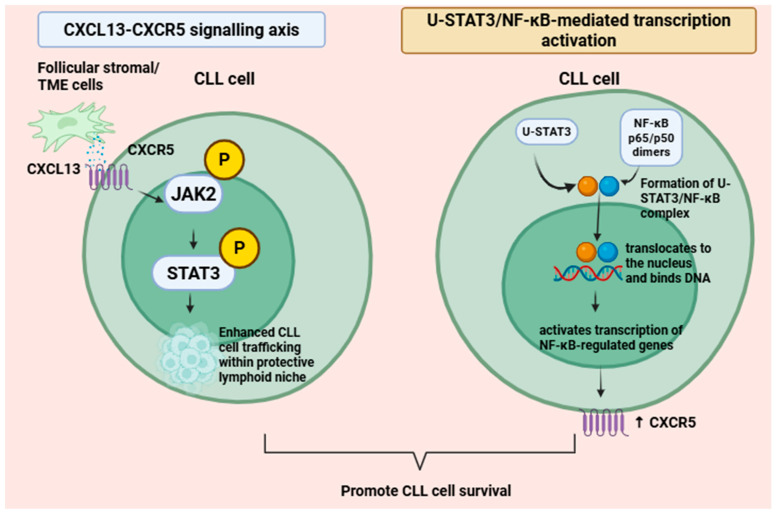
**U-STAT3/NF-κB-mediated CXCR5 upregulation drives CLL progression through coordinated tumour-promoting and immunosuppressive mechanisms**. U-STAT3/NF-κB complex formation may induce CXCR5 transcription in CLL cells, enhancing their responsiveness to CXCL13 secreted by tumour microenvironment stromal cells, which promotes CLL cell survival, proliferation, and lymphoid tissue homing [23,24,67,68]. Created in BioRender; https://BioRender.com (accessed on 23 May 2026).

Regulation of CXCR5 expression involves a complex transcriptional network comprising BCL6, BLIMP-1, NF-κB, and potentially STAT3. BCL6, the master transcriptional regulator of germinal centre differentiation, maintains CXCR5 expression by repressing B lymphocyte-induced maturation protein-1 (BLIMP-1), thereby preserving the follicular B-cell programme [66]. Constitutive activation of NF-κB, a characteristic feature of CLL, further promotes CXCR5 transcription through B-cell receptor-dependent and independent signalling pathways [67]. Emerging evidence also suggests functional interactions between unphosphorylated STAT3 (U-STAT3) and NF-κB. Rather than acting independently, U-STAT3 forms transcriptionally active complexes with NF-κB that regulate a distinct subset of target genes, including CXCR5 (Figure 2) [24,67].

Importantly, the evidence supporting this mechanism is derived primarily from experimental systems outside primary CLL cells. Consequently, whether U-STAT3 directly regulates CXCR5 transcription in CLL remains uncertain. Nevertheless, these observations provide a biologically plausible framework through which inflammatory signalling may converge on the CXCR5 homing programme.

Cytokines abundant within pseudofollicles, particularly IL-21, activate canonical STAT3 signalling and may further reinforce this transcriptional network through interactions with BCL6. However, direct experimental evidence demonstrating STAT3-dependent regulation of CXCR5 in primary CLL cells is currently lacking.

Taken together, the available evidence supports a model in which STAT3 represents a candidate regulator of the CXCR5 transcriptional programme rather than a confirmed master regulator. Experimental validation using chromatin immunoprecipitation, promoter-reporter assays, CRISPR-mediated gene perturbation, and primary CLL samples will be essential to determine whether STAT3 directly controls CXCR5 expression in vivo.

## 3. STAT3 as a Shared Molecular Integrator of Dual Chemokine Homing Axes

### 3.1. STAT3 Activation in CLL

#### 3.1.1. Constitutive JAK2/STAT3 Activation

STAT3 occupies a central position within the signalling network that regulates survival, migration, and immune interactions in CLL. Rather than functioning as an isolated signalling molecule, STAT3 integrates multiple extracellular stimuli derived from the tumour microenvironment, including inflammatory cytokines, B-cell receptor signalling, and stromal cell interactions.

Severin et al. [25] demonstrated that constitutive phosphorylation of STAT3 at Tyr705 is present in primary CLL cells and correlates with adverse clinical features, including progressive disease, cytopenias, and advanced Rai stage. Furthermore, co-culture of primary CLL cells with bone marrow mesenchymal stromal cells significantly increased STAT3 Tyr705 phosphorylation, confirming that microenvironmental signals contribute directly to STAT3 activation [25].

Importantly, different STAT3 phosphorylation sites appear to reflect distinct biological contexts. Phosphorylation of Tyr705 predominates within tissue microenvironments characterised by abundant cytokine signalling, whereas Ser727 phosphorylation is more closely associated with chemokine receptor signalling, particularly downstream of CXCR4 activation. In addition, acetylation of STAT3 at Lys685 contributes to sustained transcriptional activity and has been detected constitutively in primary CLL cells [21,25].

Collectively, these observations identify STAT3 as a convergence point through which multiple microenvironment-derived signals coordinate tumour cell survival and tissue adaptation.

#### 3.1.2. Cytokine-Mediated STAT3 Activation

Within the CLL microenvironment, cytokines provide one of the principal mechanisms driving persistent STAT3 activation. Among these, IL-6, IL-10, and IL-21 have emerged as the dominant upstream activators linking inflammatory signalling to tissue homing and tumour survival.

IL-6, produced predominantly by bone marrow stromal cells, activates the IL-6 receptor/gp130 complex, resulting in JAK1- and JAK2-dependent phosphorylation of STAT3 at Tyr705 [21,69]. Elevated circulating IL-6 concentrations in patients with CLL further support the biological relevance of this pathway.

IL-10 reinforces STAT3 activation through an autocrine and paracrine feedback mechanism. Following CXCL12-induced IL-10 secretion by CLL cells, IL-10 activates STAT3 in both malignant cells and neighbouring immune cells, thereby amplifying immune suppression within the tumour microenvironment [26,69].

In contrast, IL-21 is produced primarily by T follicular helper cells within lymph node pseudofollicles. Through activation of JAK1 and TYK2, IL-21 induces robust STAT3 phosphorylation and promotes transcriptional programmes associated with follicular homing, survival, and proliferation [26,69].

Taken together, these cytokines establish complementary signalling networks that converge upon STAT3, thereby integrating inflammatory signalling arising from anatomically distinct tissue niches.

### 3.2. STAT3 as a Direct Transcriptional Regulator of CXCR4

Among the two chemokine receptors discussed in this review, the evidence supporting STAT3-mediated regulation of CXCR4 is comparatively robust. Experimental studies have demonstrated that pharmacological inhibition of STAT3 reduces CXCR4 expression and impairs CXCL12-directed migration, suggesting that STAT3 contributes directly to regulation of the CXCR4 homing programme [25,26].

In CLL cell lines, inhibition of STAT3 using Stattic or cucurbitacin reduces surface CXCR4 expression and diminishes CXCL12-induced chemotaxis [26]. Similarly, IL-6-mediated activation of STAT3 in primary CLL cells is accompanied by increased CXCR4 surface expression, an effect that is abolished by JAK inhibition before IL-6 stimulation [25]. Together, these findings indicate that inflammatory cytokines and chemokine signalling cooperate to reinforce CXCR4 expression.

Within the bone marrow, hypoxia provides an additional regulatory layer. Activation of hypoxia-inducible factor-1α (HIF-1α) increases CXCR4 transcription, while concurrent activation of STAT3 may further enhance this response through cooperative interactions at the CXCR4 promoter [21,36]. However, the precise molecular mechanisms underlying this interaction remain incompletely defined in primary CLL cells.

Collectively, the available evidence supports a model in which STAT3 amplifies CXCR4-dependent tissue homing by integrating cytokine signalling with transcriptional regulation of CXCR4. Although additional mechanistic studies are warranted, particularly using chromatin immunoprecipitation and promoter-reporter assays in primary CLL cells, the STAT3-CXCR4 relationship is substantially better supported than the corresponding evidence for CXCR5.

### 3.3. Potential Regulation of the CXCR5 Homing Programme by STAT3

Compared with the CXCR4 axis, considerably less evidence supports direct regulation of CXCR5 by STAT3 in CLL. Nevertheless, several independent observations suggest that STAT3 may influence the CXCR5 homing programme through interactions with established transcriptional regulators.

One proposed mechanism involves unphosphorylated STAT3 (U-STAT3), which forms transcriptionally active complexes with NF-κB. These complexes regulate a distinct subset of target genes that includes CXCR5 [24,67]. Because constitutive NF-κB activation is a hallmark of CLL, this pathway provides a biologically plausible mechanism through which STAT3 may contribute to maintenance of CXCR5 expression.

Additional indirect evidence arises from the IL-21/STAT3/BCL6 signalling axis. IL-21, a cytokine abundantly produced within lymph node pseudofollicles, activates canonical STAT3 signalling and promotes BCL6-dependent transcriptional programmes associated with follicular differentiation [70]. Since BCL6 is a recognised regulator of CXCR5 expression, interactions between STAT3 and BCL6 may provide an additional route through which STAT3 influences the follicular homing phenotype.

Importantly, these mechanisms remain largely inferential in the context of CLL. Direct experimental demonstration that STAT3 binds the CXCR5 promoter or transcriptionally regulates CXCR5 expression in primary CLL cells is currently lacking. Consequently, the proposed STAT3-CXCR5 relationship should be regarded as a mechanistic hypothesis supported by indirect evidence rather than as an established signalling pathway.

Future studies employing chromatin immunoprecipitation sequencing, CRISPR-mediated gene editing, promoter-reporter assays, and single-cell multi-omic approaches will be required to determine whether STAT3 directly regulates CXCR5 transcription in CLL.

### 3.4. STAT3-Mediated Survival Signalling Within the Tumour Microenvironment

Beyond its roles in receptor regulation, STAT3 drives a broad transcriptional survival pathway within the niche environment. The canonical STAT3 target genes BCL2, MCL1, and BCL-XL are upregulated in CLL cells co-cultured with BMSCs in a JAK2/STAT3-dependent manner [25,71]. This STAT3-driven BCL-2 family upregulation directly reduces apoptotic priming of CLL cells, lowering their sensitivity to the BH3-mimetic venetoclax [31,72]. CLL cells within the BM achieve a higher apoptotic threshold than their circulating counterparts, explaining the discordance between peripheral blood response and residual nodal/marrow disease frequently observed in clinical trials [73].

Furthermore, even cells mobilised out of the niche by CXCR4 antagonism may retain elevated BCL-2 levels for a period determined by BCL-2 protein turnover, requiring concurrent venetoclax treatment to achieve cell death during the window of niche exclusion [74]. STAT3-mediated BCL-2 family upregulation within the niche thus creates a therapeutically significant disconnect between intrinsic apoptotic capacity and BH3 profiling measurements obtained from circulating cells.

Collectively, these findings indicate that STAT3 contributes not only to tissue homing but also to maintenance of the anti-apoptotic state that characterises CLL cells within protective microenvironmental niches. This dual role provides a mechanistic rationale for therapeutic strategies combining STAT3 inhibition with agents that disrupt tissue retention or target BCL-2 family proteins.

### 3.5. STAT3 as a Candidate Regulator of the CXCR4/CXCL12 and CXCR5/CXCL13 Homing Axes

The studies reviewed above support a model in which STAT3 functions as a molecular point of convergence linking inflammatory cytokines, chemokine receptor signalling, and survival pathways in CLL. Although the strength of evidence differs substantially between the CXCR4 and CXCR5 axes, these observations collectively support the hypothesis that STAT3 integrates complementary microenvironmental signals originating from both BM and LN niches.

The evidence reviewed above supports a model in which STAT3 functions as a central signalling node linking inflammatory cytokines, chemokine receptor signalling, and tissue-specific microenvironmental interactions in CLL. Constitutive activation of STAT3 by cytokines such as IL-6, IL-10, and IL-21 provides a common mechanism through which inflammatory signals originating from both the bone marrow and lymph node microenvironments may converge to regulate CLL cell survival, migration, and immune evasion [21,26]. The strength of evidence, however, differs between the two chemokine axes. Direct experimental studies support a role for STAT3 in regulating CXCR4 expression and function through cytokine-dependent transcriptional activation and downstream signalling. In contrast, the proposed relationship between STAT3 and CXCR5 is supported primarily by indirect mechanistic evidence, including interactions between unphosphorylated STAT3, NF-κB, and BCL6 signalling pathways. Although these observations provide a biologically plausible framework, direct demonstration of STAT3-dependent regulation of CXCR5 in primary CLL cells remains lacking.

Taken together, these findings support the hypothesis that STAT3 acts as a candidate molecular integrator, rather than a confirmed master regulator, of the CXCR4/CXCL12 and CXCR5/CXCL13 homing axes. Through integration of cytokine signalling with chemokine receptor pathways, STAT3 may coordinate bone marrow retention, lymph node homing, immune suppression, and resistance to therapy, thereby contributing to disease progression [21,25]. Importantly, this proposed model generates several experimentally testable predictions, including whether simultaneous inhibition of STAT3 and both chemokine axes provides greater therapeutic benefit than targeting either pathway alone, and whether combined assessment of STAT3 activation together with CXCR4 and CXCR5 expression improves prognostic stratification.

**Figure 3:**
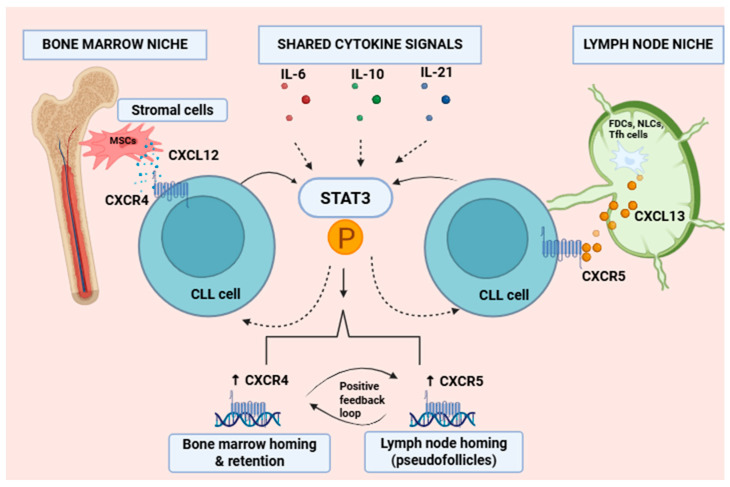
**Proposed model illustrating STAT3 as a candidate molecular integrator of the CXCR4/CXCL12 and CXCR5/CXCL13 homing axes in chronic lymphocytic leukemia (CLL).** Within the bone marrow niche, stromal cells, including mesenchymal stromal cells (MSCs), produce CXCL12, which activates CXCR4 on CLL cells. In lymph nodes, follicular dendritic cells (FDCs), nurse-like cells (NLCs), and T follicular helper (Tfh) cells produce CXCL13, promoting CXCR5-mediated homing and pseudofollicle localisation. Shared cytokines, including IL-6, IL-10, and IL-21, activate STAT3, which is proposed to integrate signalling from both tissue microenvironments. Current evidence supports STAT3-mediated regulation of CXCR4, whereas regulation of CXCR5 remains a biologically plausible but incompletely validated mechanism in primary CLL cells. Activation of STAT3 is proposed to enhance tissue homing, survival, immune suppression, therapy resistance, and disease progression through positive feedback mechanisms. Created in BioRender; https://BioRender.com (accessed on 29 June 2026).

A conceptual model summarising the proposed integration of the CXCR4/CXCL12 and CXCR5/CXCL13 homing pathways through STAT3 signalling is presented in Figure 3.

## 4. Comparative Analysis of the Dual Homing Axes in CLL

Although the CXCR4/CXCL12 and CXCR5/CXCL13 signalling axes have traditionally been investigated as independent pathways, the evidence reviewed throughout this manuscript indicates that they perform complementary rather than redundant biological functions. Together, these chemokine systems coordinate the continuous trafficking of CLL cells between the bone marrow and lymph node microenvironments, thereby allowing malignant cells to exploit the distinct survival and proliferative advantages offered by each anatomical niche.

Rather than functioning as isolated signalling pathways, the two axes appear to be integrated through shared cytokine networks and overlapping downstream signalling mechanisms. This raises the possibility that coordinated regulation of both pathways may contribute more substantially to disease progression than activation of either axis alone.

### 4.1. Tissue Compartment Specificity and Dynamic Receptor Switching

CLL cells in the peripheral blood exist in a state of ongoing recirculation between BM and SLO compartments. Calissano et al. [40] established that circulating CLL cells segregate into CXCR4^dim^CD5^bright^ (proliferative) and CXCR4^bright^CD5^dim^ (resting) intraclonal fractions reflecting recent lymphoid tissue transit. Mazzarello et al. [75] subsequently demonstrated that BCR signalling capacity and co-receptor density, including IgM and HLA-DR, amplify progressively from the resting to the proliferative fraction, while CXCR4 shows the opposite pattern, with lowest surface density on proliferative cells. These observations confirm that the CXCR4/CD5 phenotypic switch encodes not merely trafficking history but a coordinated shift in BCR signalling competence across the intraclonal gradient. The molecular basis for this dynamic switching likely involves the differential internalisation kinetics of the two receptors.

The phenotypic distinction between these subpopulations has been substantially refined by subsequent work. Friedman et al. [41] demonstrated that the CXCR4^dim^CD5^bright^ fraction co-expresses high surface IgM, CXCR5, and CCR7, and exhibits efficient chemotactic migration, consistent with recent pseudofollicular activation. Surface CXCR4 within this fraction is dynamically regulated: it is rapidly downregulated following CD40, IL-4, or CXCL12 stimulation but resurfaces when stimulation ceases or cells progress through the cell cycle. Following LN egress, most CXCR4^dim^CD5^bright^ emigrants revert to a quiescent CXCR4^hi^CD5^lo^ state with the lowest surface IgM and diminished BCR calcium flux, consistent with G0 arrest. Coelho et al. [76] demonstrated complementary intraclonal heterogeneity in IgM density and BCR signalling capacity, confirming that functional BCR competence varies dynamically with niche exposure. Bartholdy et al. [42] demonstrated that the two fractions carry distinct DNA methylation landscapes reflecting their proliferative histories, with the proliferative fraction enriched for epigenetic marks of disease progression. Collectively, the CXCR4/CD5 phenotypic switch represents not merely a surface marker of recent LN transit but an index of durable niche-instilled epigenetic programming, underscoring the imperative for therapeutic strategies that prevent niche re-entry rather than targeting circulating cells alone.

### 4.2. Shared vs. Divergent Downstream Signalling

Although the two chemokine axes operate within anatomically distinct microenvironments, they converge upon several common downstream signalling pathways (Table 1). Among these, STAT3 occupies a unique position because it integrates inflammatory cytokine signalling, chemokine receptor activity, and transcriptional programmes associated with migration, survival, and immune suppression.

PI3Kδ is a shared node; both CXCR4- and CXCR5-mediated chemotaxis in CLL are sensitive to PI3K inhibition, establishing PI3Kδ as a common transducer of both chemokine signals and a rational target for simultaneous axis disruption [77]. Both receptors also activate ERK1/2, though the sustained nature of CXCR5-induced ERK1/2 phosphorylation appears to be a more prominent feature of CXCR5 signalling in CLL than of CXCR4 [41,54,78]. At the STAT3 level, CXCR4 is specifically linked to pS727-STAT3 induction and the downstream IL-10 immunosuppressive axis, whereas CXCR5 drives pY705-STAT3 indirectly through the IL-21/Tfh circuit within the pseudofollicle [26,55]. These two STAT3 phosphorylation events may have distinct transcriptional outputs, though their relative contributions to CXCR4/CXCR5 transcriptional regulation have not been dissected experimentally.

### 4.3. Clinical Correlates of the Two Axes

CXCR4-high expression is associated with advanced Rai stage, higher absolute lymphocyte counts, and greater BM infiltration by trephine biopsy [13,45,48,49]. Elevated serum CXCL13 is associated with lymphadenopathic disease, more frequent splenomegaly, and greater propensity for Richter’s transformation [20,54]. Patients with high serum CXCL13 have shorter TTT and, in preliminary datasets, inferior responses to ibrutinib monotherapy. Patients who exhibit simultaneously high CXCR4 expression and elevated serum CXCL13 likely represent the most treatment-resistant subgroup, possessing parallel mechanisms for BM retention and LN proliferative activity. This hypothesis has not been directly tested in a prospectively designed clinical study, a gap that represents a high priority for future investigation and a limitation of the current evidence base.

## 5. Therapeutic Implications: Disrupting Both Axes

### 5.1. Ibrutinib

Ibrutinib produces a characteristic transient lymphocytosis during the first weeks of treatment, reflecting rapid egress of CLL cells from bone marrow and secondary lymphoid tissue niches into the peripheral blood [79,80]. Mechanistically, BTK inhibition disrupts BCR-CXCR4 crosstalk at several levels. It inhibits BTK-dependent phosphorylation of CXCR4, impairs WASp-mediated actin reorganisation required for CXCL12-directed migration, and reduces CXCR4-dependent stromal adhesion and pseudoemperipolesis [28,44,81].

Ibrutinib may also attenuate STAT3-dependent niche signalling. Ex vivo studies have shown that ibrutinib reduces Ser727 phosphorylation of STAT3 in CLL cells, suggesting partial disruption of the CXCR4-STAT3 signalling axis [28]. Furthermore, combined inhibition of BTK and JAK2/STAT3 signalling produces greater CLL cell death in stromal co-culture models than BTK inhibition alone, indicating that STAT3 contributes to stromal-mediated cytoprotection [50].

Whether CLL cells that develop resistance to covalent BTK inhibitors re-engage CXCR4- or CXCR5-dependent niche homing remains unresolved. The C481S BTK mutation partially restores BCR signalling, but its effect on chemokine receptor function, STAT3 activation, and microenvironmental dependence has not been fully defined. Available evidence suggests that ibrutinib suppresses CXCL12-, CXCL13-, and CCL19-induced adhesion and migration of primary CLL cells and remodels macrophage-CLL and stromal interactions [44,81,82]. Additionally, Kennedy et al. [83] showed that Toll-like receptor 9 (TLR9) signaling promotes CLL cell migration and tissue homing via NF-κB and STAT3 activation, suggesting an alternative microenvironmental pathway that may sustain disease despite BTK inhibition. Therefore, loss of BTK inhibitor activity may allow partial restoration of tissue homing pathways, but this remains a hypothesis requiring prospective validation in samples from patients progressing on BTK inhibitors.

### 5.2. PI3Kδ Inhibitors

PI3Kδ inhibitors, including idelalisib, induce rapid lymph node shrinkage accompanied by redistribution lymphocytosis, a clinical pattern consistent with disruption of lymphoid tissue retention [60,84]. Mechanistically, PI3Kδ inhibition potently suppresses CXCL13-directed CLL cell migration, supporting the importance of PI3Kδ downstream of CXCR5 signalling [85]. Although PI3Kδ inhibition is most closely linked to disruption of lymph node homing, it may also affect CXCR4-mediated chemotaxis because CXCR4 signalling uses PI3Kδ as well as PI3Kγ. This overlap suggests that PI3Kδ inhibitors may partially disrupt both lymph node and bone marrow trafficking pathways [86]. However, the relative contribution of CXCR5 versus CXCR4 inhibition to the clinical redistribution effect remains difficult to separate and requires further mechanistic study.

### 5.3. Venetoclax and Niche-Mediated BCL-2 Upregulation

Venetoclax induces deep remissions in CLL by selectively inhibiting BCL-2, but microenvironmental protection can attenuate venetoclax-induced apoptosis. Co-culture with bone marrow stromal cells reduces CLL cell sensitivity to venetoclax through JAK2/STAT3-dependent upregulation of MCL-1 and BCL-XL [87]. This finding suggests that STAT3 activation within protective niches may increase the apoptotic threshold of CLL cells and thereby reduce dependence on BCL-2 alone.

Residual disease following venetoclax therapy is frequently enriched within bone marrow and lymph node compartments, consistent with the concept that tissue niches provide survival signals that limit complete eradication [88]. Therefore, combining venetoclax with agents that mobilise CLL cells from protective niches, such as BTK inhibitors or CXCR4 antagonists, represents a biologically plausible strategy. However, the optimal timing, sequence, and patient population for such combinations remain to be defined clinically.

### 5.4. Direct CXCR4 Antagonism

Direct targeting of CXCR4 represents a rational strategy for disrupting bone marrow retention. AMD3100 (plerixafor), a small-molecule CXCR4 antagonist, mobilises CLL cells from the bone marrow into the peripheral blood, reduces CXCL12-directed migration, and disrupts pseudoemperipolesis in stromal co-culture models [45]. The CXCR4 inhibitor T140 has been shown to suppress CXCL12-induced STAT3 and MAPK phosphorylation in CLL cells, inhibiting their activity and migration in bone marrow stroma [17]. These effects demonstrate that CXCR4 is functionally important for maintaining CLL cell interactions with the protective marrow niche.

Nevertheless, CXCR4 antagonism alone is unlikely to be sufficient as a durable therapeutic strategy because mobilisation does not necessarily induce cell death. Instead, CXCR4 blockade may be most useful as a sensitising approach when combined with cytotoxic, targeted, or pro-apoptotic agents. This strategy requires further clinical validation, particularly in relation to optimal scheduling with venetoclax or BTK inhibitors.

### 5.5. Immunomodulatory Disruption of the CXCR4-STAT3-IL-10 Axis

The CXCL12-CXCR4-STAT3-IL-10 axis provides a mechanistic link between tissue homing and immune suppression in CLL. Lenalidomide has been shown to reduce basal and CXCL12-induced Ser727 phosphorylation of STAT3 in CLL cells, accompanied by reduced IL-10 production [26]. These findings suggest that immunomodulatory therapy may disrupt not only tumour–microenvironment interactions but also the immunosuppressive signalling pathways downstream of CXCR4.

However, the clinical use of lenalidomide in CLL is limited by toxicity and has largely been superseded by more effective targeted therapies. Its relevance in this context is therefore primarily mechanistic: it demonstrates that pharmacological disruption of the CXCR4-STAT3-IL-10 axis can reduce CLL-mediated immune suppression.

### 5.6. STAT3 Inhibitors in CLL

STAT3 inhibition represents an attractive investigational strategy because STAT3 integrates cytokine signalling, survival pathways, and, at least for CXCR4, chemokine receptor regulation. Direct STAT3 inhibitors, including Stattic and cucurbitacin, induce apoptosis in CLL models and reduce expression of downstream survival genes such as BCL2 and MCL1 [25]. JAK inhibitors, including ruxolitinib and AG490, block upstream STAT3 phosphorylation and have provided proof-of-concept evidence for targeting this pathway [89].

However, therapeutic claims regarding STAT3 inhibition should be made cautiously. Current evidence does not yet establish that STAT3 inhibitors will be most effective in patients co-expressing high levels of CXCR4 and CXCR5, nor has this biomarker-guided strategy been tested prospectively. In addition, the role of STAT3 in CXCR5 regulation remains incompletely validated in primary CLL cells. Therefore, STAT3 inhibition should be framed as a promising experimental approach that requires further preclinical and clinical evaluation, rather than as an established dual-axis therapeutic strategy.

### 5.7. Therapeutic Synthesis

Collectively, current therapeutic evidence supports the principle that disrupting CLL cell interactions with tissue microenvironments can enhance treatment responses. BTK inhibitors, PI3Kδ inhibitors, venetoclax combinations, CXCR4 antagonists, and STAT3-directed approaches each interfere with different components of this protective network. However, direct evidence supporting simultaneous therapeutic targeting of the CXCR4/CXCL12, CXCR5/CXCL13, and STAT3 pathways remains limited.

The most balanced interpretation is that dual-axis disruption represents a rational and testable therapeutic concept. Future studies should determine whether combined targeting of chemokine-mediated homing and STAT3-dependent survival signalling improves depth of response, reduces residual disease within bone marrow and lymph node niches, and delays emergence of therapy resistance.

## 6. Discussion

### 6.1. The Unified Model: Contribution and Implications

The principal contribution of this review is the formulation of a unified conceptual model in which STAT3 is proposed to function as a candidate molecular integrator of microenvironment-derived cytokine signalling and the CXCR4/CXCL12 and CXCR5/CXCL13 homing axes in CLL. While CXCR4, CXCR5, and STAT3 signalling have each been reviewed extensively in isolation, to our knowledge no previous review has integrated these pathways into a single framework that links inflammatory cytokine signalling, tissue homing, immune modulation, and therapeutic resistance while generating experimentally testable mechanistic hypotheses.

The integration of the CXCL12-CXCR4-STAT3-IL-10 immunosuppressive axis [26], the clinically validated CXCL13/galectin-9 biomarker panel [59], and evidence that JAK2/STAT3 inhibition can overcome bone marrow stromal cell-mediated resistance to ibrutinib [25] substantially strengthens the biological plausibility of this framework. Nevertheless, the strength of evidence differs across the proposed pathways. Whereas the STAT3–CXCR4 relationship is supported by experimental studies in CLL models, the proposed STAT3–CXCR5 axis remains based primarily on indirect mechanistic evidence and therefore requires direct validation in primary CLL cells. Accordingly, the unified model presented here should be regarded as a hypothesis-driven framework rather than a definitive mechanistic pathway.

From a therapeutic perspective, these observations suggest that disrupting a single component of the microenvironmental network may be insufficient to achieve durable disease control. Ibrutinib promotes mobilisation of CLL cells from protective tissue niches and induces partial regression of lymphadenopathy; however, minimal residual disease frequently persists within the bone marrow and lymph node microenvironments, where cytokine-mediated activation of STAT3 and complementary homing pathways may continue to support CLL cell survival [79,80]. Similarly, venetoclax monotherapy may leave residual CLL cells within tissue niches despite achieving deep peripheral blood responses [88]. Although these observations support the rationale for targeting multiple microenvironmental pathways simultaneously, whether combined disruption of STAT3 and both chemokine axes provides superior clinical benefit remains to be established in prospective studies.

### 6.2. Limitations of the Published Literature

Interpretation of the current literature on CXCR4 and CXCR5 in CLL is limited by considerable methodological heterogeneity. Flow cytometric assessment of receptor expression has been reported using mean fluorescence intensity (MFI), mean fluorescence intensity ratio (MFIR), percentage-positive cells, or estimated receptor number per cell, often employing different antibody clones, gating strategies, and instrument settings. Standardised reporting guidelines, together with the use of calibrated bead standards to convert fluorescence intensity into antibody-binding capacity, would substantially improve the comparability and reproducibility of future studies. The prospective study by Xue et al. [49] demonstrated that simultaneous assessment of CXCR4 expression during routine CLL immunophenotyping is technically feasible and provides clinically relevant prognostic information. Similarly, Karimi et al. [53] highlighted methodological variability in CXCR4 quantification as an important contributor to inconsistent findings across studies, an observation that is equally applicable to CXCR5 and underscores the need for harmonised analytical approaches.

A second limitation is the heavy reliance on immortalised CLL cell lines. Much of the mechanistic evidence supporting STAT3-mediated regulation of chemokine signalling has been generated using MEC-1, OSU-CLL, or EHEB cell lines, which harbour additional genetic alterations and may not accurately recapitulate the transcriptional and signalling landscape of primary CLL cells. Consequently, direct validation in primary patient-derived samples remains essential.

Finally, the proposed STAT3-centred model has yet to be evaluated using emerging technologies such as single-cell RNA sequencing, spatial transcriptomics, and chromatin immunoprecipitation sequencing (ChIP-seq). These complementary approaches offer an opportunity to determine whether STAT3 directly regulates CXCR4 and CXCR5 transcription within anatomically distinct tissue niches and whether the proposed signalling network operates uniformly across the malignant clone or is restricted to specific CLL subpopulations. Such studies will be critical for distinguishing experimentally validated mechanisms from biologically plausible hypotheses.

## 7. Conclusions

The CXCR4/CXCL12 and CXCR5/CXCL13 axes represent two anatomically distinct yet functionally complementary chemokine pathways that regulate CLL cell trafficking between the bone marrow and lymph node microenvironments. The evidence reviewed in this manuscript supports the hypothesis that STAT3 may function as a candidate molecular integrator linking inflammatory cytokine signalling with these complementary homing pathways. While direct evidence supporting STAT3-mediated regulation of CXCR4 is comparatively strong, the proposed interaction with CXCR5 remains incompletely validated in primary CLL cells and therefore requires further experimental investigation.

Collectively, the available data support a model in which cytokine-driven STAT3 activation contributes to tissue homing, immune modulation, and survival signalling within protective CLL niches. This framework provides a biologically plausible explanation for the persistence of residual disease within tissue compartments despite the efficacy of contemporary targeted therapies in clearing circulating CLL cells. Importantly, the model presented here should be regarded as a hypothesis-driven conceptual framework that integrates current evidence while identifying key gaps for future investigation.

Recent clinical observations demonstrating the prognostic value of CXCL13 and galectin-9, the ability of JAK2/STAT3 inhibition to overcome bone marrow stromal cell-mediated resistance, and the reversibility of the CXCL12-CXCR4-STAT3-IL-10 immunosuppressive pathway further support the translational relevance of this model. If validated experimentally, simultaneous targeting of STAT3 together with complementary chemokine-mediated homing pathways may represent a rational strategy to overcome microenvironment-mediated survival and therapeutic resistance in CLL. Ultimately, defining whether STAT3 functions as the shared molecular interface between bone marrow retention and lymph node homing has the potential to advance our understanding of CLL biology and guide the development of future microenvironment-directed therapeutic strategies.

## Figures and Tables

**Table 1:** Comparative overview of the CXCR4/CXCL12 and CXCR5/CXCL13 homing axes in chronic lymphocytic leukemia (CLL).

Feature	CXCR4/CXCL12	CXCR5/CXCL13
Primary tissue	Bone marrow	Lymph node
Major ligand source	CAR cells, MSCs	FDCs, NLCs, Tfh cells
Major signalling	PI3K/AKT, JAK2/STAT3	PI3Kδ, ERK/MAPK
Clinical phenotype	Bone marrow infiltration	Lymphadenopathy
Dynamic regulation	Highly dynamic (CXCR4^dim^CD5^bright^ vs. CXCR4^bright^CD5^dim^)	Relative stable surface expression
Current evidence linking STAT3	Direct	Indirect
Prognostic association	Shorter PFS, more advanced disease	Shorter TTFT, PFS, OS

**Abbreviations**: MSCs, mesenchymal stromal cells; FDCs, follicular dendritic cells; NLCs, nurse-like cells; Tfh, T follicular helper cells; PI3K, phosphoinositide 3-kinase; ERK, extracellular signal-regulated kinase; STAT3, signal transducer and activator of transcription 3; TTFT, Time to First Treatment; PFS, Progression-Free Survival; OS, Overall Survival.

## Data Availability

All data supporting this review are available within the article.
